# First Demonstration of Hysteresis-Free IGZO/SnO-Based Complementary Circuits and SRAM with Long-Term Reliability Using SU-8 Passivation

**DOI:** 10.1186/s40580-025-00517-x

**Published:** 2025-10-31

**Authors:** Changwoo Han, Hyeonjung Park, Yejoo Choi, Myeongjae Choi, Jaehyuk Lim, Huiseong Shin, Seungjoon Moon, Changhwan Shin

**Affiliations:** 1 School of Electrical Engineering, Korea University, Seoul, 02841 South Korea; 2 Department of Electrical and Computer Engineering, Sungkyunkwan University, Suwon, 16419 South Korea; 3 Semiconductor R&D Center, Samsung Electronics, Hwaseong, 18448 South Korea; 4 Department of Semiconductor System Engineering, Korea University, Seoul, 02841 South Korea; 5 Research Institute of Semiconductor Technology, Korea University, Seoul, 02841 South Korea

**Keywords:** Oxide semiconductor, Thin-film transistor (TFT), Indium gallium zinc oxide (IGZO), Tin monoxide (SnO), Inverter, Ring oscillator (RO), Static random-access memory (SRAM)

## Abstract

Complementary logic and memory circuits based on n-type indium gallium zinc oxide (IGZO) and p-type tin monoxide (SnO) thin-film transistors (TFTs) were demonstrated with low-voltage, hysteresis-free operation. Optimization of IGZO channel thickness precisely tuned the inverter switching point to near V_DD_/2, achieving a high voltage gain of 146.6 V/V at V_DD_ = 3 V and ultra-low static power consumption in the nanowatt range. SU-8 passivation effectively suppressed bias-stress-induced degradation in both IGZO and SnO TFTs, enhancing long-term stability and reducing device variation. Using these optimized devices, a 3-stage ring oscillator exhibited stable oscillations, and 6T-SRAM cells achieved tunable static noise margins by adjusting transistor strength ratios. This work represents one of the first implementations of IGZO/SnO-based  6T-SRAM and demonstrates the potential of oxide semiconductor complementary circuits for low-power and reliable system integration, with promising future applicability to non-volatile memory and on-device artificial intelligence hardware.

**Graphical abstract:**

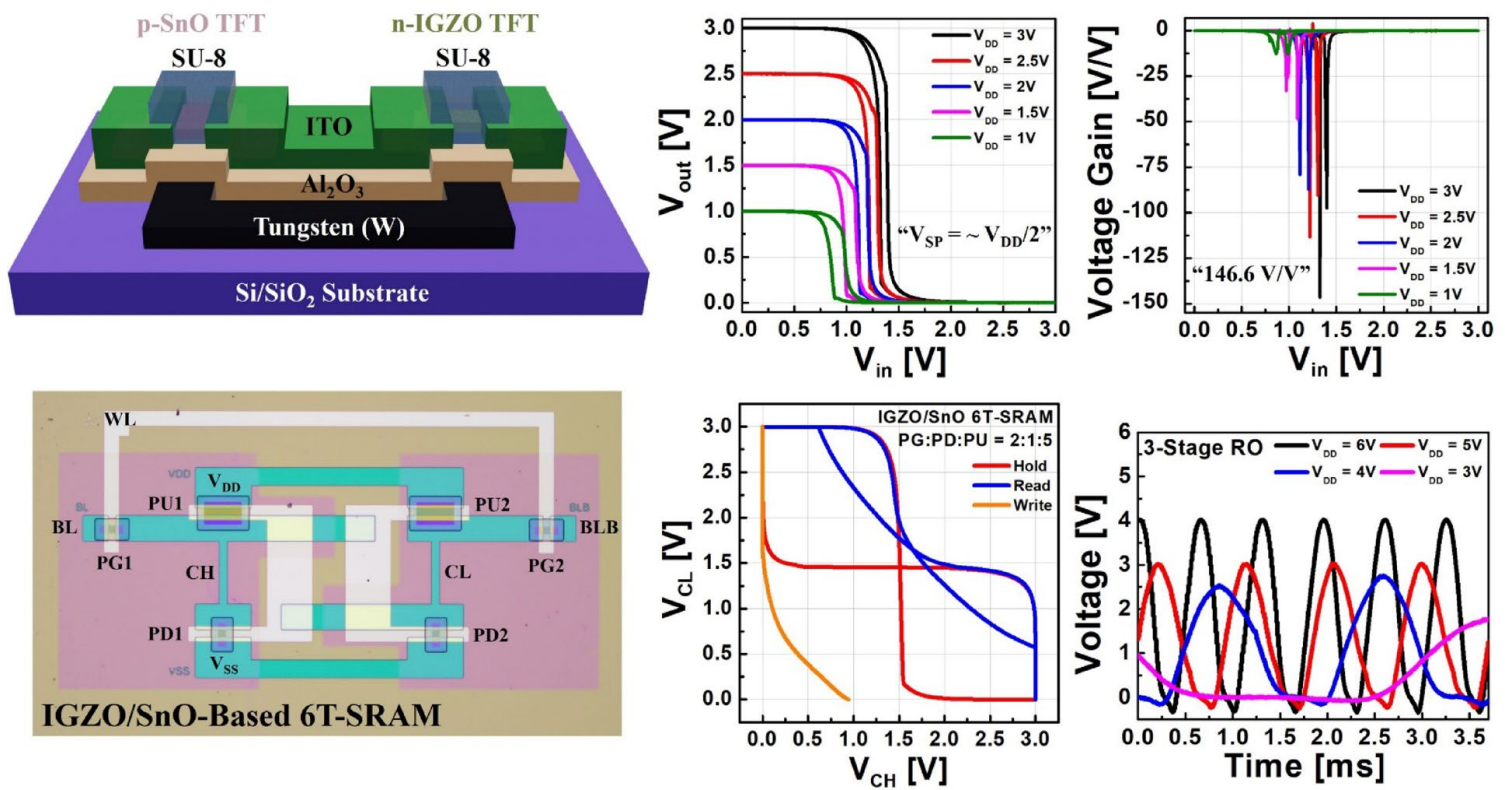

## Introduction

Oxide semiconductors have attracted significant attention across various fields, including next-generation displays, sensors, wearable electronics, and low-power logic applications, due to their unique advantages such as high mobility, wide bandgap, low-temperature processing compatibility, optical transparency, and mechanical flexibility for implementation on flexible substrates [1–4]. Among these materials, n-type oxide semiconductors, particularly indium-gallium-zinc oxide (IGZO), have already achieved widespread commercial adoption owing to their well-established thin-film transistor (TFT) fabrication technologies and excellent electrical and process stability. IGZO TFTs are now extensively utilized in OLED display drivers, sensor interfaces, and analog signal amplification circuits, demonstrating their high mobility and reliable operation under various operating conditions.

In stark contrast, the development of p-type oxide semiconductors continues to face critical challenges that have limited their practical implementation. Fundamental issues such as intrinsically low hole mobility, high subgap trap density, and unstable surface conditions have significantly hindered their integration into complementary circuits [5, 6]. These challenges stem from the inherent electronic structure of most oxide materials, where the valence band maximum is typically composed of highly localized oxygen 2p orbitals, resulting in poor hole transport properties. Nonetheless, tin monoxide (SnO) has recently emerged as one of the most promising p-type oxide semiconductors due to its unique delocalized 5 s valence band structure, which enables relatively high hole mobility and sharp switching characteristics compared to conventional p-type oxides. The ability to form crystalline SnO at low processing temperatures, combined with its stable p-type conduction mechanism, makes it an attractive candidate for integration with IGZO in complementary logic architectures. However, SnO-based devices remain highly sensitive to processing conditions, including oxygen partial pressure, oxidation states, and thermal treatment parameters. Furthermore, critical reliability concerns such as hysteresis effects, environmental degradation under humidity and oxygen exposure, and long-term stability under prolonged electrical stress continue to pose significant challenges that must be systematically addressed for practical circuit applications.

Given these fundamental material challenges, complementary inverters that effectively combine n-type and p-type oxide TFTs are essential for enabling low-power operation, high voltage gain, and wide noise margins in oxide-based logic circuits. Achieving full rail-to-rail output swing in digital building blocks such as ring oscillators, SRAM cells, and NAND gates requires precisely balanced electrical characteristics between the n-type and p-type devices, particularly in terms of drive current, threshold voltage, and switching speed. Recently, significant research efforts have been devoted to developing oxide–semiconductor-based inverters for low-power logic applications [7–17]. However, most prior works have suffered from critical limitations, including high operating voltages (typically > 5 V), low voltage gain (< 20 V/V), asymmetric voltage transfer characteristics (VTCs), limited output voltage swing, and the presence of hysteresis under bidirectional voltage sweep conditions. These performance limitations significantly constrain their scalability for practical large-scale integrated circuit applications.

Moreover, comprehensive studies that include systematic assessments of long-term electrical reliability or the effects of repeated bias stress remain relatively scarce in the literature. Given the stringent power constraints of emerging wearable and portable electronic systems, circuits capable of stable operation at low supply voltages (≤ 3 V) are highly desirable for practical implementation. To achieve such low-voltage operation while maintaining robust performance, several critical requirements must be met: precise performance matching between n-type and p-type devices, accurate threshold voltage (V_th_) tuning to optimize switching characteristics, and enhanced tolerance against process-induced device variations. Among various approaches to address these challenges, effective passivation strategies have emerged as crucial for mitigating electrical degradation and improving device stability. In this context, SU-8 polymer passivation has demonstrated particular promise due to its excellent compatibility with low-temperature processing requirements, inherent mechanical flexibility for bendable applications, and superior environmental barrier properties against moisture and oxygen ingress [18, 19].

In this study, we present a comprehensive investigation of complementary logic and memory circuits based on n-type IGZO TFTs and p-type SnO TFTs, including inverters, ring oscillators, and six-transistor static random-access memory (6 T-SRAM) architectures. SU-8 passivation layers were systematically implemented to suppress bias-stress-induced degradation, and the resulting devices demonstrated excellent long-term reliability under rigorous testing conditions, including prolonged DC voltage sweeping and negative/positive bias temperature instability (NBTI/PBTI) stress evaluations. Most notably, stable hysteresis-free logic inversion was successfully achieved at a low operating voltage of 3 V, as rigorously confirmed through bidirectional VTC measurements, with the optimized inverters demonstrating an exceptionally high voltage gain of up to 146.6 V/V. Furthermore, systematic threshold voltage tuning through IGZO channel thickness optimization enabled significant improvements in static noise margin (SNM) for robust SRAM operation. Through careful adjustment of transistor strength ratios within the memory cell architecture, we conducted comprehensive analysis of the impact on read/write characteristics and successfully identified optimal design conditions for enhanced SRAM stability and performance.

These experimental results collectively highlight the substantial practical potential of IGZO/SnO-based complementary circuits for future system-level integration in advanced electronic applications. The demonstrated low-voltage operation, high reliability, and excellent circuit performance establish this material platform as a promising foundation for next-generation low-power, highly reliable digital systems. Moreover, the proven compatibility with flexible processing and the demonstrated circuit functionality provide a clear pathway for extension toward integration with emerging technologies, including non-volatile memory architectures and energy-efficient on-device artificial intelligence (AI) hardware platforms, thereby opening new opportunities for ubiquitous computing applications.

## Methods

**Fig. 1:**
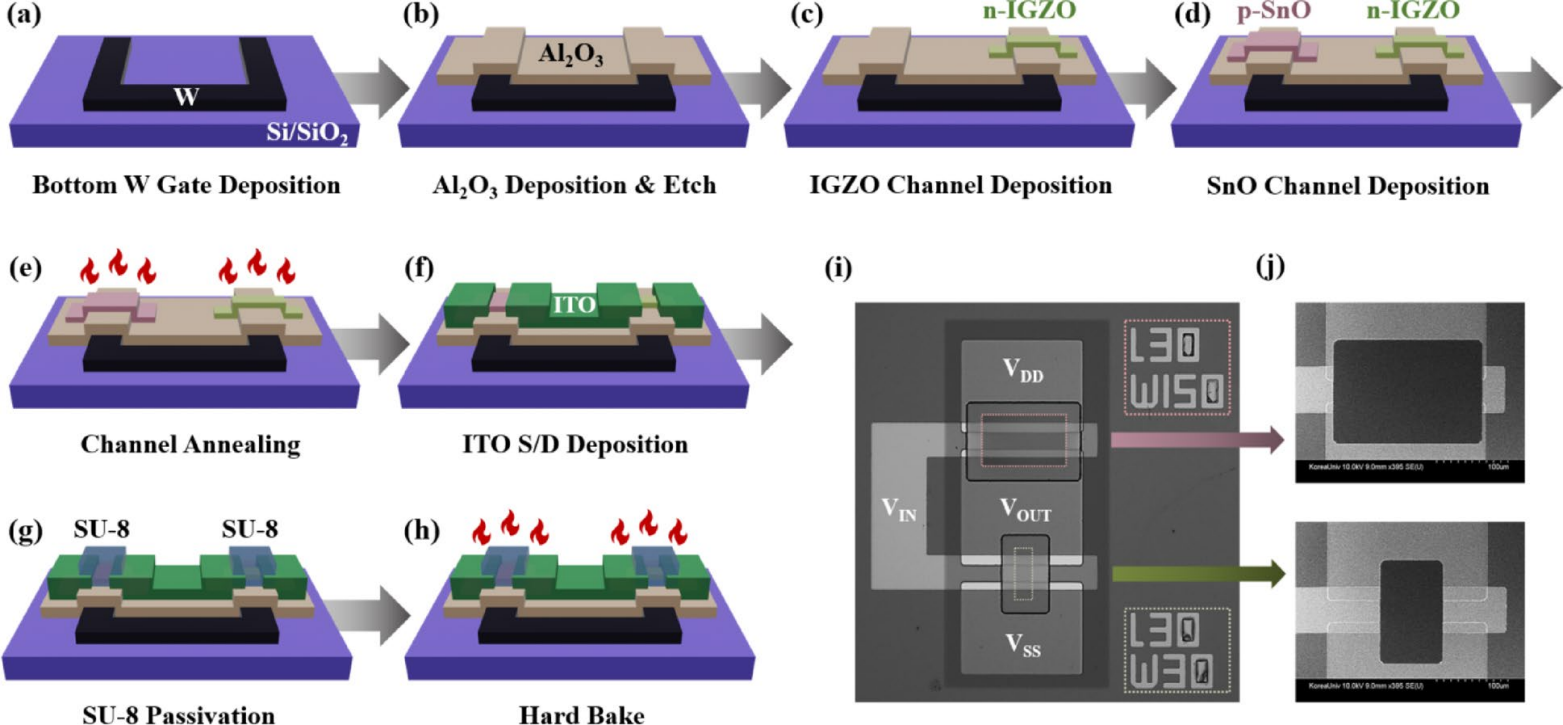
**a**-**h** Schematic of the fabrication process of IGZO/SnO complementary inverter. **i** Optical microscope image of the fabricated complementary inverter and **j** Scanning electron microscope images of the IGZO and SnO channels

The complete fabrication process flow of the IGZO/SnO complementary inverter is illustrated in Fig. 1a–h. Initially, a Si/SiO_2_ substrate was cleaned and prepared, followed by photoresist (PR) coating for negative lithography to define the gate electrode pattern. Tungsten (W) gate electrodes were deposited by DC magnetron sputtering for 550 s (resulting in ~ 60 nm thickness) and subsequently patterned using a lift-off process in acetone, leaving only the defined gate electrode regions (Fig. 1a). A 10 nm-thick Al_2_O_3_ gate dielectric layer was then deposited by atomic layer deposition (ALD) at 250 °C using trimethylaluminum (TMA) and H_2_O as precursors with a deposition rate of approximately 0.12 nm/cycle. Positive photolithography was performed to define the gate dielectric regions, followed by selective etching in buffered oxide etchant (BOE) to remove unwanted Al_2_O_3_, and the remaining photoresist was subsequently removed with acetone (Fig. 1b).

For the n-type channel formation, negative photolithography was conducted to define the IGZO channel regions, and IGZO thin films (In_2_O_3_:Ga_2_O_3_:ZnO = 1:1:1 mol%) were deposited by RF magnetron sputtering for 960 s (resulting in ~ 30 nm thickness) at room temperature under an Ar atmosphere, followed by lift-off processing (Fig. 1c). The p-type channel was fabricated through RF magnetron sputtering of SnO for 120 s (resulting in ~ 14 nm thickness) using a metallic Sn target in an Ar/O_2_ gas mixture, followed by lift-off to precisely define the channel geometry (Fig. 1d). Negative lithography was deliberately employed for both IGZO and SnO channel patterning to avoid potential plasma damage to the underlying Al_2_O_3_ dielectric layer during conventional etching processes. Following channel deposition, both IGZO and SnO films were simultaneously annealed at 300 °C for 10 min on a hot plate in ambient air to promote crystallization and activate the semiconductor properties (Fig. 1e).

Source/drain (S/D) electrodes were patterned using negative photolithography, and indium tin oxide (ITO, In_2_O_3_:SnO_2_ = 9:1 mol%) contact layers were deposited by RF magnetron sputtering for 900 s at room temperature, followed by lift-off processing to form the final electrode structure (Fig. 1f). Since the bottom-gate TFT architecture inherently leaves the active channel regions exposed to ambient air, SU-8 polymer passivation was applied as a protective encapsulation layer against environmental degradation from oxygen and moisture ingress. SU-8 photoresist was spin-coated at 4000 rpm for 30 s to achieve uniform coverage, followed by positive photolithography to selectively pattern the passivation layer to cover only the IGZO and SnO channel regions while leaving contact pads accessible. The patterned SU-8 layer was finally hard-baked at 150 °C for 30 min to enhance cross-linking density and optimize the barrier properties of the passivation layer (Fig. 1g–h). The optical microscope image of a representative fabricated complementary inverter is shown in Fig. 1i, while Fig. 1j presents high-resolution scanning electron microscope images of the IGZO and SnO channel regions, respectively. The identical fabrication procedure was employed for all circuit demonstrations, including ring oscillator and 6 T-SRAM architectures.

Comprehensive electrical characterization of the fabricated devices was performed using a semiconductor parameter analyzer (Keithley 4200-SCS) under ambient conditions at room temperature. For ring oscillator measurements, a high-speed digital oscilloscope (Keysight DSOX1204G) was employed to capture rapid voltage transitions and analyze oscillation frequency characteristics. SRAM cell measurements were conducted using an additional precision DC power supply (Keysight EDU36311A) to provide independent bias control for comprehensive read/write operation and stability testing. Structural and morphological characterization was carried out using Cs-corrected scanning transmission electron microscopy (STEM, JEM-ARM200F), optical microscopy (OM, Olympus BX53M) for device layout verification, and scanning electron microscopy (SEM, HITACHI S-4800) for surface morphology analysis. The chemical composition and oxidation states of the IGZO and SnO active layers were systematically analyzed by X-ray photoelectron spectroscopy (XPS, Thermo Fisher Scientific K-Alpha +).

## Results and discussion

### Electrical characteristics of SnO and IGZO TFTs

**Fig. 2:**
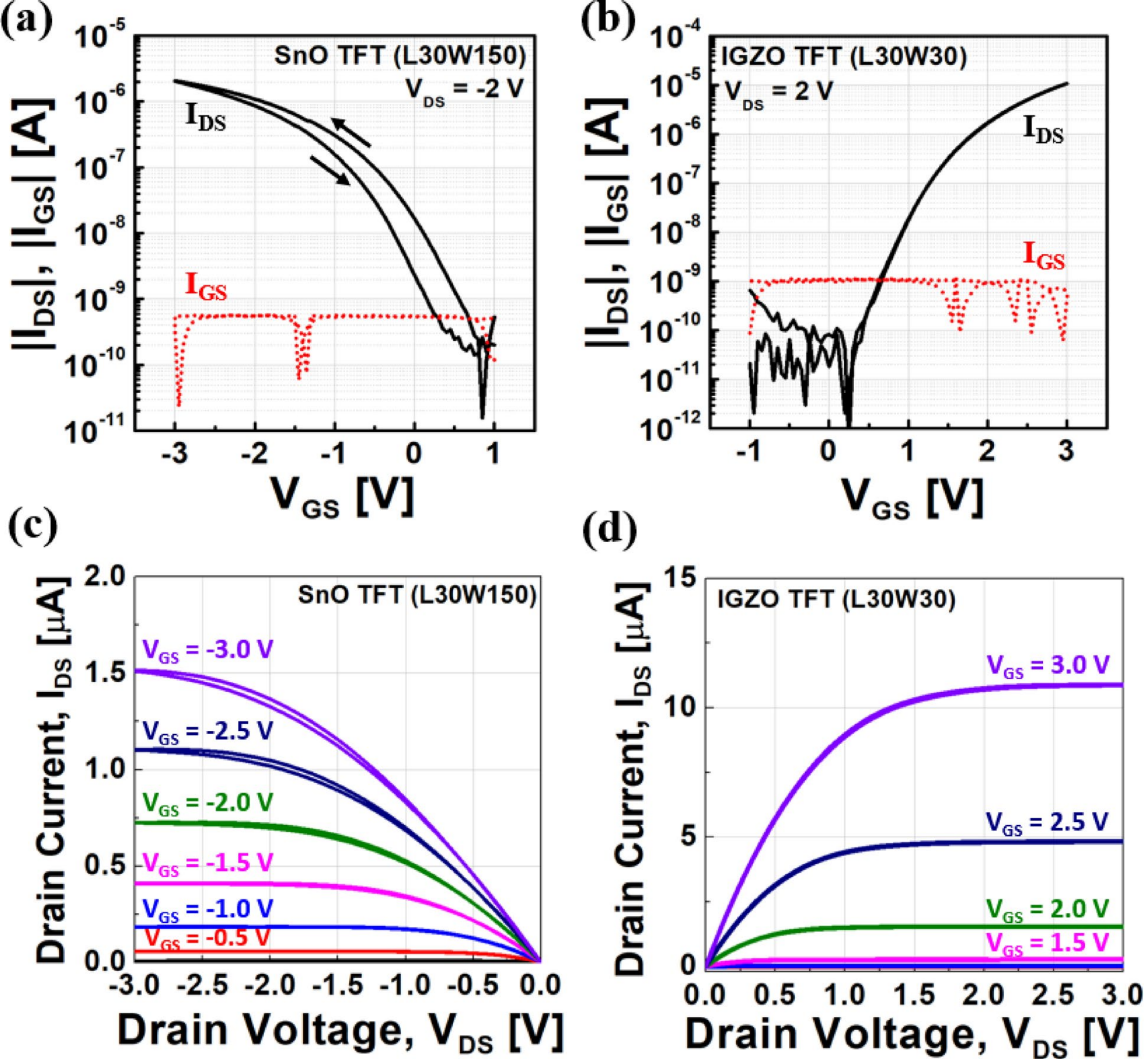
**a**, **b** Tranfer characteristics of the fabricated p-type SnO TFT (L = 30 μm, W = 150 μm) at V_DS_ =  − 2 V and n-type IGZO TFT (L = 30 μm, W = 30 μm) at V_DS_ = 2 V, respectively. **c **,**d** Output characteristics of the fabricated p-type SnO TFT and n-type IGZO TFT for various V_GS_, respectively

The transfer and output characteristics of the fabricated p-type SnO TFT (L = 30 μm, W = 150 μm) and n-type IGZO TFT (L = 30 μm, W = 30 μm) are shown in Fig. 2. Both forward and reverse I_DS_-V_GS_ and I_DS_-V_DS_ sweeps were conducted to evaluate hysteresis behavior, and the V_th_ was extracted at I_DS_ = 10^–8^ A using the constant current method.

For the SnO TFT, the forward sweep yielded a V_th_ of 0.22 V, an on/off current ratio of 1.05 × 10^4^, a subthreshold swing (SS) of 355.51 mV/dec, and a field-effect mobility of 0.1087 cm^2^/V∙s. In the reverse sweep, V_th_ shifted to − 0.18 V, with an on/off current ratio of 3.96 × 10^3^, SS of 327.93 mV/dec, and mobility of 0.1847 cm^2^/V∙s. Notably, compared to previously reported p-type oxide TFTs, the hysteresis was significantly reduced, enabling stable p-type conduction while minimizing potential performance degradation under repeated operation. The output characteristics exhibit typical p-type transistor behavior, with a linear increase in drain current at low drain voltages followed by current saturation at higher drain voltages, indicating good ohmic contact formation between the ITO electrode and the SnO channel.

For the IGZO TFT, the forward sweep resulted in a V_th_ of 0.96 V, an on/off current ratio of 1.72 × 10^5^, SS of 187.44 mV/dec, and mobility of 8.8619 cm^2^/V∙s. The reverse sweep maintained the same V_th_ (0.96 V), while the on/off current ratio increased to 9.75 × 10^5^, SS improved to 160.75 mV/dec, and mobility was 8.3466 cm^2^/V∙s. The output characteristics display clear linear and saturation regions, confirming good ohmic contact between the ITO electrode and the IGZO channel along with excellent field-effect behavior.

**Fig. 3:**
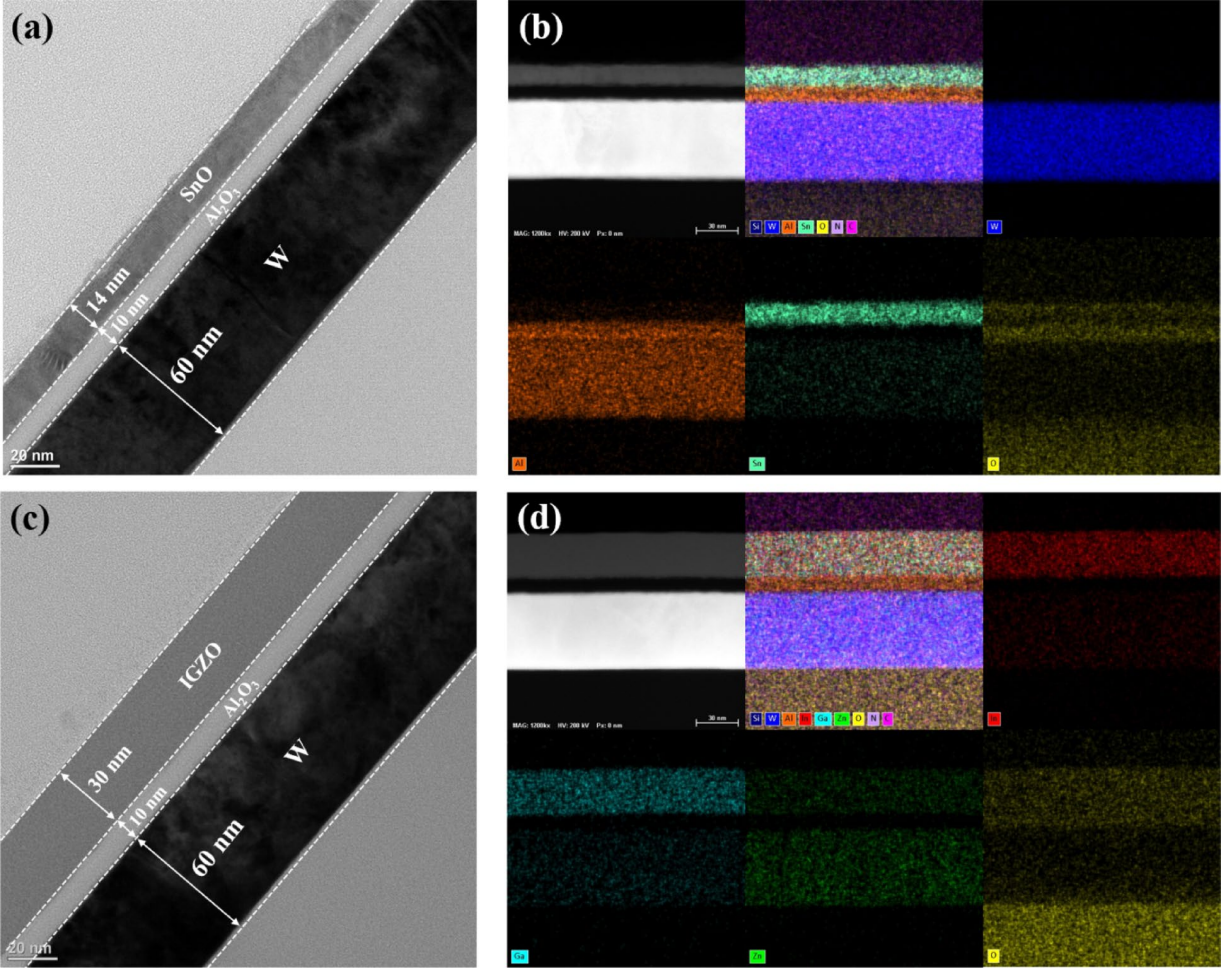
**a**, **b** Cross-sectional TEM image and corresponding EDS elemental mapping of the p-type SnO TFT, showing a 60 nm W gate, 10 nm Al_2_O_3_ gate dielectric, and 14 nm SnO channel. **c**, **d** Cross-sectional TEM image and corresponding EDS elemental mapping of the n-type IGZO TFT, showing a 60 nm W gate, 10 nm Al_2_O_3_ gate dielectric, and 30 nm IGZO channel. The EDS results confirm uniform film deposition and well-defined interfaces

Cross-sectional TEM images (Fig. 3) reveal a well-defined bottom-gate structure consisting of a 60 nm W gate electrode, a 10 nm Al_2_O_3_ gate dielectric, a 14 nm SnO channel, and a 30 nm IGZO channel. Energy-dispersive X-ray spectroscopy (EDS) elemental mapping confirmed uniform film deposition and well-defined layer interfaces without significant interdiffusion.

**Fig. 4:**
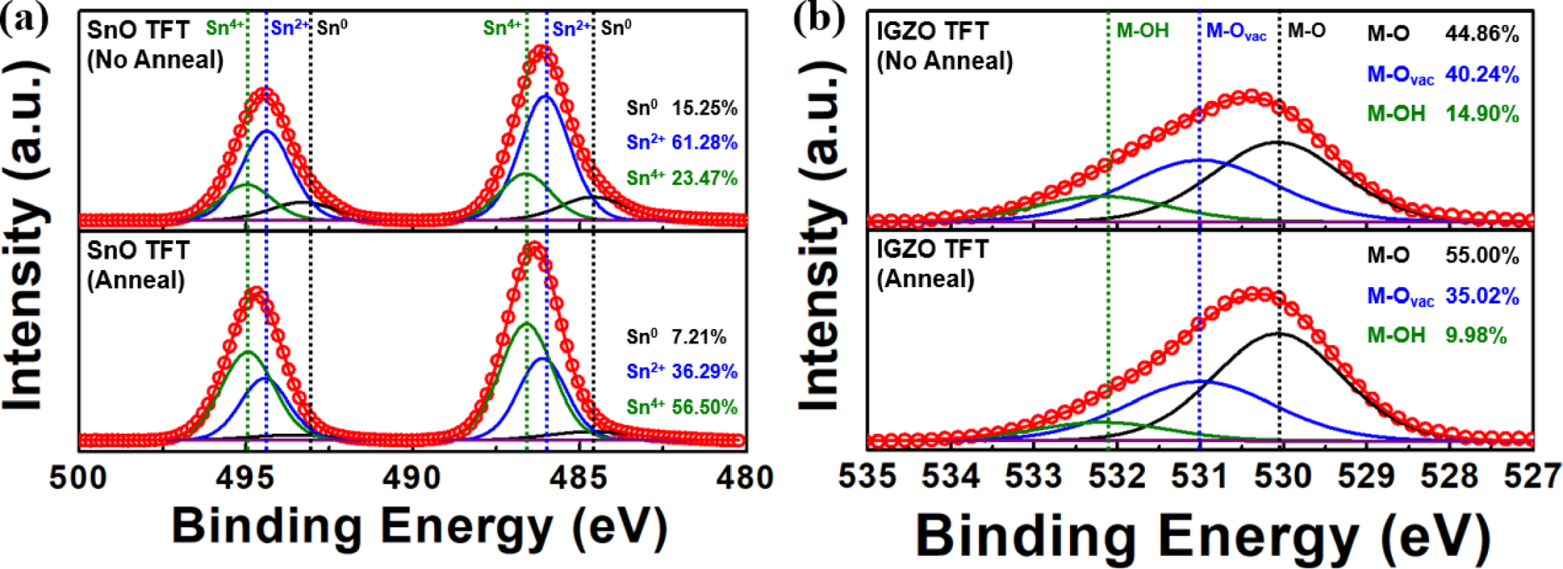
XPS spectra of **a** Sn 3d core levels in the p-type SnO TFT and **b** O 1 s core levels in the n-type IGZO TFT, before and after annealing

XPS analysis was conducted to investigate chemical composition and oxidation state changes in the channel layers. In the SnO TFT (Fig. 4a), the Sn 3d_5/2_ peaks were observed at 484.60 eV (Sn^0^), 486.04 eV (Sn^2+^), and 486.60 eV (Sn^4+^), while the Sn 3d_3/2_ peaks appeared at 493.30 eV (Sn^0^), 494.39 eV (Sn^2+^), and 494.99 eV (Sn^4+^), consistent with values reported in previous studies [20–22]. After annealing, the metallic Sn^0^ content decreased significantly from 15.25 to 7.21%, while Sn^2+^ decreased from 61.28 to 36.29%, and Sn^4+^ increased from 23.47 to 56.50%. Although the increase in Sn^4+^ may potentially be unfavorable for p-type conduction, the substantial reduction of metallic Sn^0^, which is known to disrupt p-type conduction paths and cause electrical instability [21, 22], plays a critical role in enhancing channel stability and device reproducibility.

For the IGZO TFT (Fig. 4b), the O 1 s spectrum shows metal–oxygen (M–O) bonds at 530.08 eV, metal–oxygen vacancy (M-O_vac_) bonds at 531.00 eV, and metal-hydroxyl lattice (M-OH) bonds at 532.19 eV, which agree well with literature values [16, 23, 24]. After annealing, the proportion of M–O bonds increased from 44.86 to 55.00%, while M-O_vac_ and M-OH bonds decreased from 40.24 to 35.02% and from 14.90 to 9.98%, respectively. The increase in M–O bonds is expected to improve electrical stability by extending carrier transport paths and reducing defect density within the channel.

Overall, comprehensive electrical, structural, and chemical analyses confirm that both SnO and IGZO TFTs exhibit low hysteresis, stable output characteristics, and improved channel quality after thermal annealing, providing a robust foundation for the fabrication of complementary circuits.

### IGZO/SnO-based inverter characteristics

**Fig. 5:**
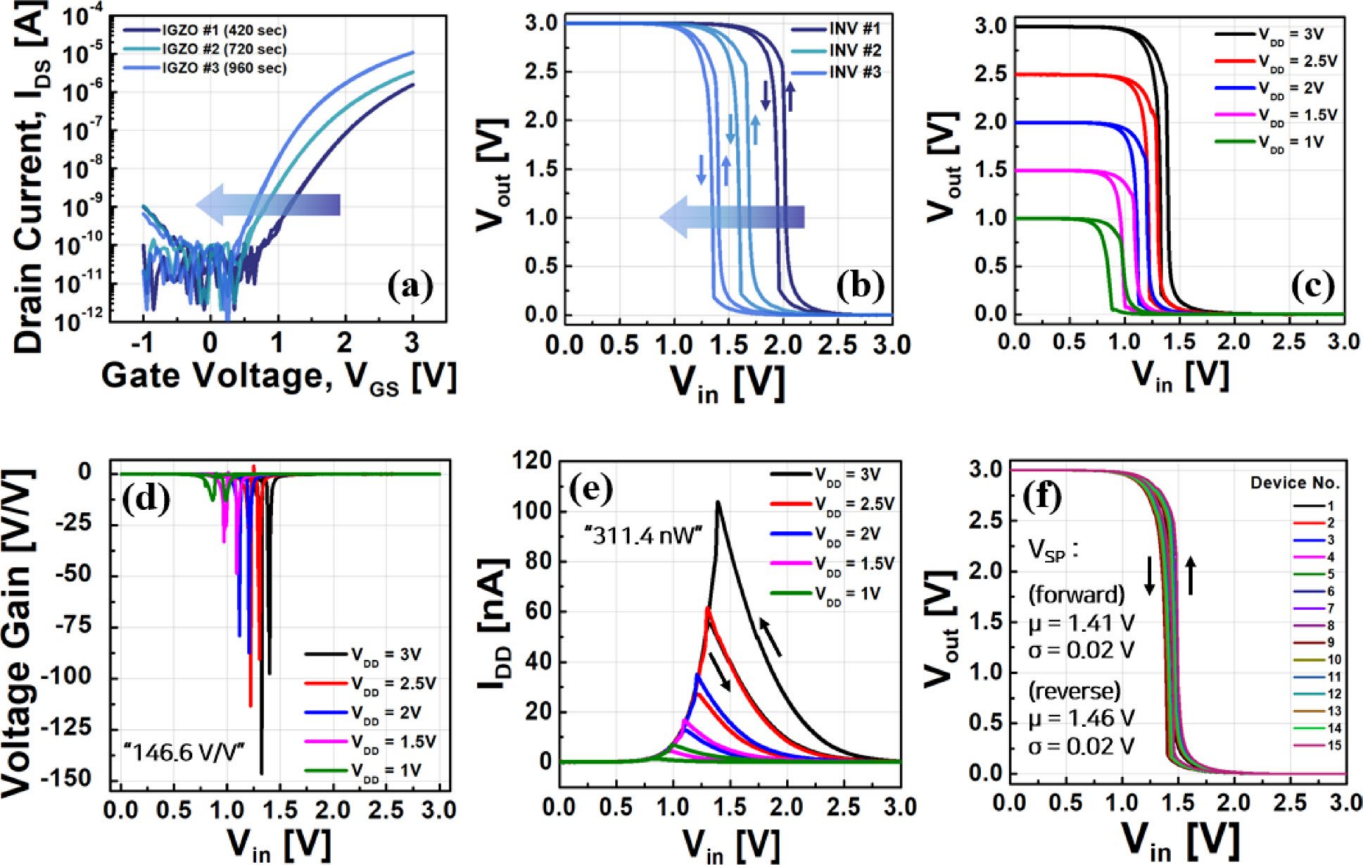
Electrical characteristics of the fabricated IGZO/SnO-based complementary inverters. **a** Transfer characteristics of IGZO TFTs with varying channel deposition times (420, 720, and 960 s). **b** Corresponding voltage transfer characteristics (VTCs) showing the shift of switching point toward V_DD_/2 with increased IGZO thickness. **c** Measured VTCs for V_DD_ ranging from 1 to 3 V. **d** Voltage gain characteristics, with a maximum gain of 146.6 V/V at V_DD_ = 3 V. **e** Current transfer characteristics showing extremely low static power consumption (P_max_) values for different V_DD_. **f** Device-to-device variation in switching point voltage (V_SP_), showing excellent reproducibility with standard deviations of 0.02 V in both forward and reverse sweeps

The characteristics of the fabricated IGZO/SnO-based inverters are shown in Fig. 5. Figure 5a presents the electrical characteristics of IGZO TFTs with varying channel thickness. Following previous optimization studies [25, 26], the IGZO channel deposition time was systematically adjusted to 420 s (IGZO #1), 720 s (IGZO #2), and 960 s (IGZO #3), and the V_th_ was found to shift negatively as the channel thickness increased. This adjustment was intentionally made to optimize the voltage transfer characteristic (VTC) curve of the inverter toward V_DD_/2, as shown in Fig. 5b. For instance, INV #1, with a thinner IGZO channel, exhibits a switching point above V_DD_/2, which is unfavorable for securing adequate noise margins. In contrast, INV #3, with an increased channel thickness, shows a switching point near V_DD_/2, thus ensuring sufficient noise margins for reliable logic operation. Based on these optimization results, INV #3 was selected as the final device configuration.

In this inverter design, the n-type IGZO channel has dimensions of L = 30 μm and W = 30 μm, resulting in an aspect ratio of 1, whereas the p-type SnO channel has dimensions of L = 30 μm and W = 150 μm, giving an aspect ratio of 5. This asymmetric design choice compensates for the inherently lower drive capability of the p-type channel by increasing its channel width, which plays a crucial role in achieving high voltage gain even at low operating voltages.

Table 1 summarizes the switching point voltage (V_SP_), voltage gain, and maximum static power consumption (P_max_) values for each V_DD_. Figure 5c shows the measured VTC curves for V_DD_ ranging from 1 to 3 V. At V_DD_ = 3 V, the V_SP_ is 1.32 V in the forward sweep and 1.39 V in the reverse sweep, both close to the ideal V_DD_/2 (1.5 V), ensuring stable noise margins. The noise margin high (NM_H_) and noise margin low (NM_L_) were 1.47 V (97.9% of V_DD_/2) and 1.18 V (78.5% of V_DD_/2), respectively. As shown in Fig. 5d, the maximum voltage gain (-dV_out_/dV_in_) reaches 146.6 V/V at V_DD_ = 3 V, which is among the highest reported for oxide-based inverters operating at low supply voltages. As summarized in Table 2, previously reported oxide-based complementary inverters typically require relatively high supply voltages and demonstrate modest gains with limited static noise margins. Such high gain at low voltages is advantageous for low-power circuit design, providing strong noise immunity and stable logic operation.

**Table 1:** Electrical parameters of IGZO/SnO-based inverters at different supply voltages

V_DD_ [V]	V_SP_ [V]		|Gain| [V/V]		P_max_ [nW]	
	Forward	Forward	Forward	Reverse	Forward	Reverse
3	1.32	1.39	146.60	97.65	165.66	311.40
2.5	1.21	1.30	113.50	90.56	67.93	153.73
2	1.11	1.20	79.21	87.50	25.32	69.90
1.5	0.97	1.08	33.24	48.73	6.71	24.96
1	0.80	0.89	12.81	13.14	1.39	6.93

**Table 2:** Summary of recent studies on oxide-based complementary inverters

Channel materials	V_DD_ [V]	Gain [V/V]	NM_H_ [V]	NM_L_ [V]	Year
n-IGZO p-SnO_x_	17	4.2	6.5	0.6	2011 [7]
n-ZnO p-SnO	12	12	6.2	3.8	2016 [8]
n-IGZO p-SnO	40	24	20	14.4	2017 [9]
n-IGZO p-SnO	10	92.4	–	–	2019 [10]
n-IGZO p-Cu_x_O	20	14	–	–	2020 [11]
n-ZTO p-Cu_2_O	20	4.2	7	6.5	2020 [12]
n-IGZO p-SnO_x_	10	33.4	4.4	4.7	2022 [13]
n-IGZO p-SnO	70	240	–	–	2023 [14]
n-IGZO p-Cu_2_O	20	14	–	–	2023 [15]
n-IGTO p-SnO_x_	10	114.5	3.9	4.5	2023 [16]
n-IGZO p-SnO	12	54.1	–	–	2024 [17]
n-IGZO p-SnO	3	146.6	1.47	1.18	This work

The current transfer curves in Fig. 5e reveal extremely low P_max_ values, ranging from 1.39 to 311.4 nW for V_DD_ = 1–3 V, enabling ultra-low-power operation suitable for battery-powered IoT devices and energy harvesting systems. Finally, Fig. 5f demonstrates minimal device-to-device variation. The average V_SP_ values were 1.41 V (forward) and 1.46 V (reverse), with standard deviations of only 0.02 V in both cases, indicating excellent device reproducibility and process uniformity. Furthermore, while many previous studies on oxide-based complementary inverters did not report dual-sweep transfer characteristics, potentially overlooking the presence of hysteresis, our work provides a complete and transparent analysis of both forward and reverse V_SP_. As shown in Table 1, the difference between forward and reverse switching points is only 0.07 V at V_DD_ = 3 V, confirming the practically hysteresis-free behavior of the proposed inverter.

### Effect of SU-8 passivation

**Fig. 6:**
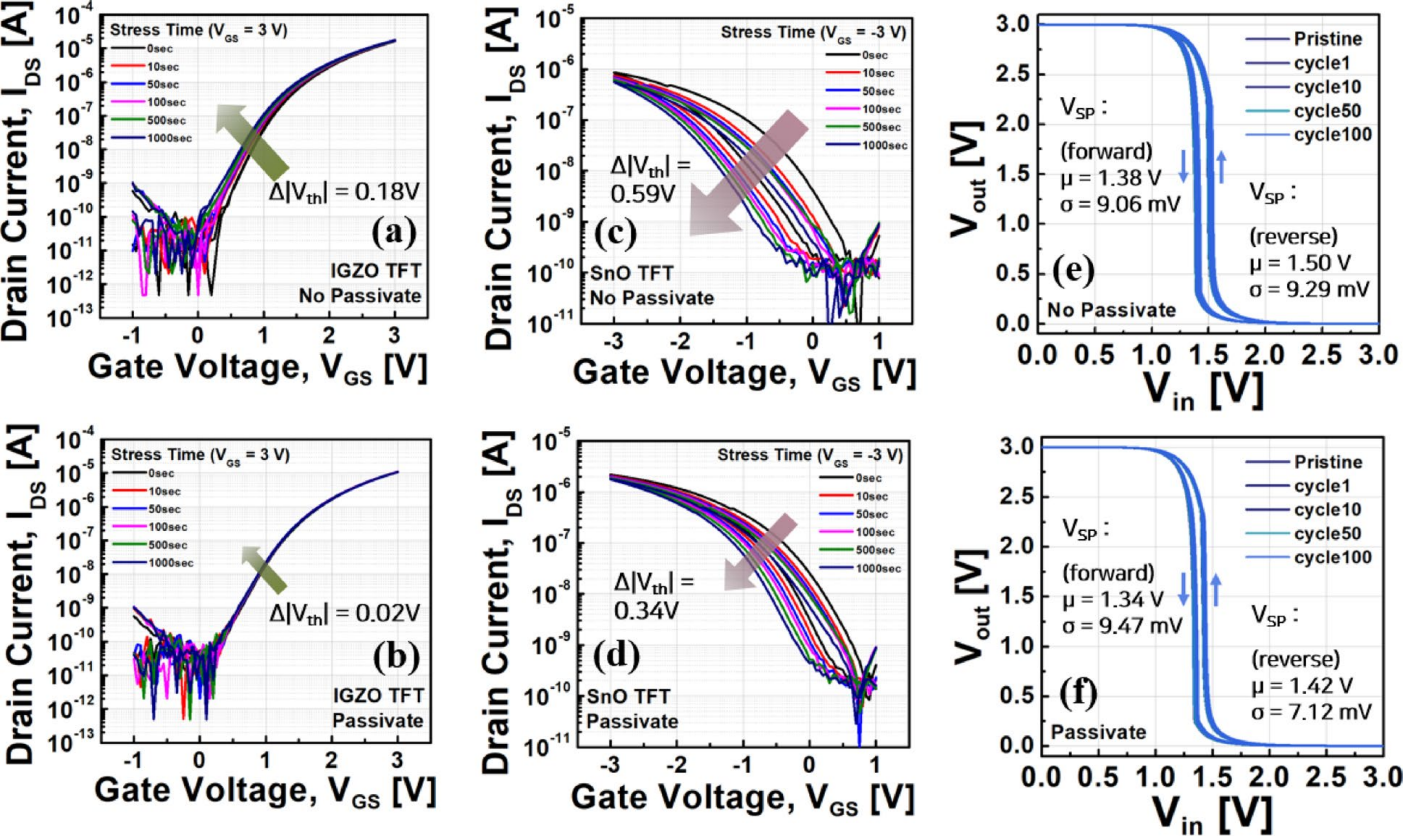
Effect of SU-8 passivation on the stability of IGZO and SnO TFTs and corresponding inverter performance. **a**, **b** Transfer characteristics of IGZO TFTs under positive bias stress (V_GS_ = 3 V) without and with SU-8 passivation, respectively. **c**, **d** Transfer characteristics of SnO TFTs under negative bias stress (V_GS_ =  − 3 V) without and with SU-8 passivation, respectively. **e**, **f** Voltage transfer characteristics (VTCs) of inverters without and with SU-8 passivation over 100 switching cycles, showing reduced variation in switching point voltage (V_SP_​) with passivation

The effect of SU-8 passivation on the stability of IGZO and SnO TFTs is shown in Fig. 6. Figure 6a and b compare the I_DS_-V_GS_ characteristics of IGZO TFTs under positive gate bias stress (V_GS_ = 3 V) over time. Without passivation (Fig. 6a), the threshold voltage shift (Δ|V_th_|) reached approximately 0.18 V after 1000 s of stress, whereas with SU-8 passivation (Fig. 6b), Δ|V_th_| was significantly reduced to 0.02 V, indicating substantially improved device stability.

A similar trend was observed for SnO TFTs under bias stress conditions. Figure 6c and d show the I_DS_-V_GS_ characteristics under negative gate bias stress (V_GS_ =  − 3 V). Without passivation (Fig. 6c), Δ|V_th_| was 0.59 V, but with SU-8 passivation (Fig. 6d), it decreased to 0.34 V. These results demonstrate that SU-8 passivation effectively suppresses channel degradation under prolonged operation, significantly enhancing long-term electrical stability.

The passivation effect was also evident in inverter performance. Figure 6e and f present the VTC variations of inverters without and with passivation, respectively. Based on the V_SP_ values, the non-passivated inverter exhibited average values of 1.38 V (forward) and 1.50 V (reverse) with standard deviations of 9.06 mV and 9.29 mV, respectively. In contrast, the passivated inverter showed average values of 1.34 V (forward) and 1.42 V (reverse) with standard deviations of 9.47 mV and 7.12 mV, respectively, indicating reduced variation particularly in the reverse sweep. This confirms that SU-8 passivation is also effective in maintaining stable operation under repeated switching cycles.

### IGZO/SnO-based ring oscillator characteristics

**Fig. 7:**
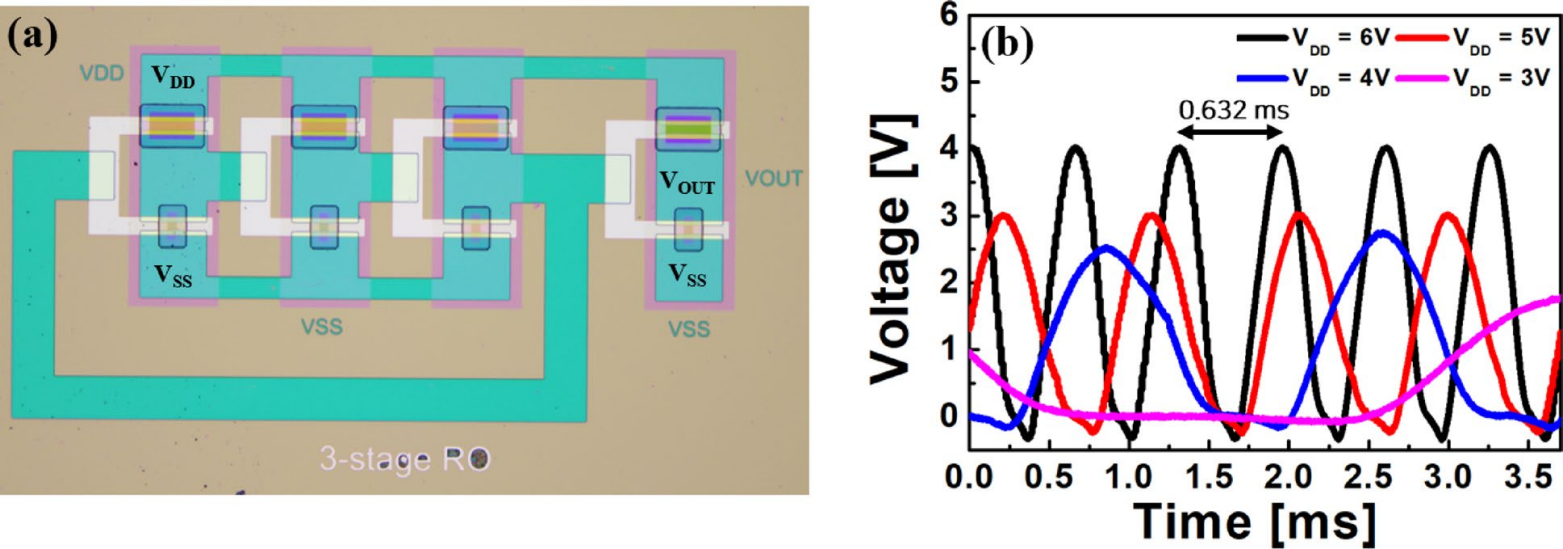
**a** Optical microscope image of the fabricated 3-stage ring oscillator (RO) with buffers using IGZO/SnO inverters. **b** Measured output waveforms of the RO for different supply voltages (V_DD_ = 3–6 V), showing stable oscillations at all voltages. At V_DD_ = 6 V, the oscillation period was 0.632 ms (1.58 kHz), corresponding to a delay time of ~ 105.33 μs per stage

A 3-stage ring oscillator (RO) with output buffers was designed using the fabricated IGZO/SnO inverters, and its performance was evaluated using a digital oscilloscope (Fig. 7). Figure 7b shows the output waveforms measured for V_DD_ ranging from 3 to 6 V. Stable oscillations were observed at all operating voltages, demonstrating that the fabricated inverters can reliably perform continuous logic switching operations. At V_DD_ = 6 V, the oscillation period was approximately 0.632 ms, corresponding to an oscillation frequency of 1.58 kHz. The delay time per stage was calculated to be ~ 105.33 μs. These results confirm that the IGZO/SnO-based inverters can achieve stable oscillation characteristics, making them suitable for use in clock generation and timing circuits.

### Noise margin characteristics of IGZO/SnO-based 6 T-SRAM

In this study, 6 T-SRAM cells based on IGZO/SnO TFTs were fabricated, and their stability was evaluated by measuring the hold static noise margin (HSNM), read static noise margin (RSNM), and write noise margin (WNM). Noise margin is a critical metric for SRAM stability, and in oxide semiconductor-based implementations, it is essential to carefully analyze performance variations caused by the intrinsic drive capability differences between n-type and p-type devices. For each operation, the SNM was defined as the side length of the largest square that can be inscribed in the corresponding voltage transfer curve loop.

The fabricated 6 T-SRAM cell consists of pass-gate (PG), pull-down (PD), and pull-up (PU) transistors, and the strength ratio between them significantly affects the cell’s read/write behavior. The channel length of all transistors was fixed at 30 μm, and the drive strength was adjusted by varying the channel width.

**Fig. 8:**
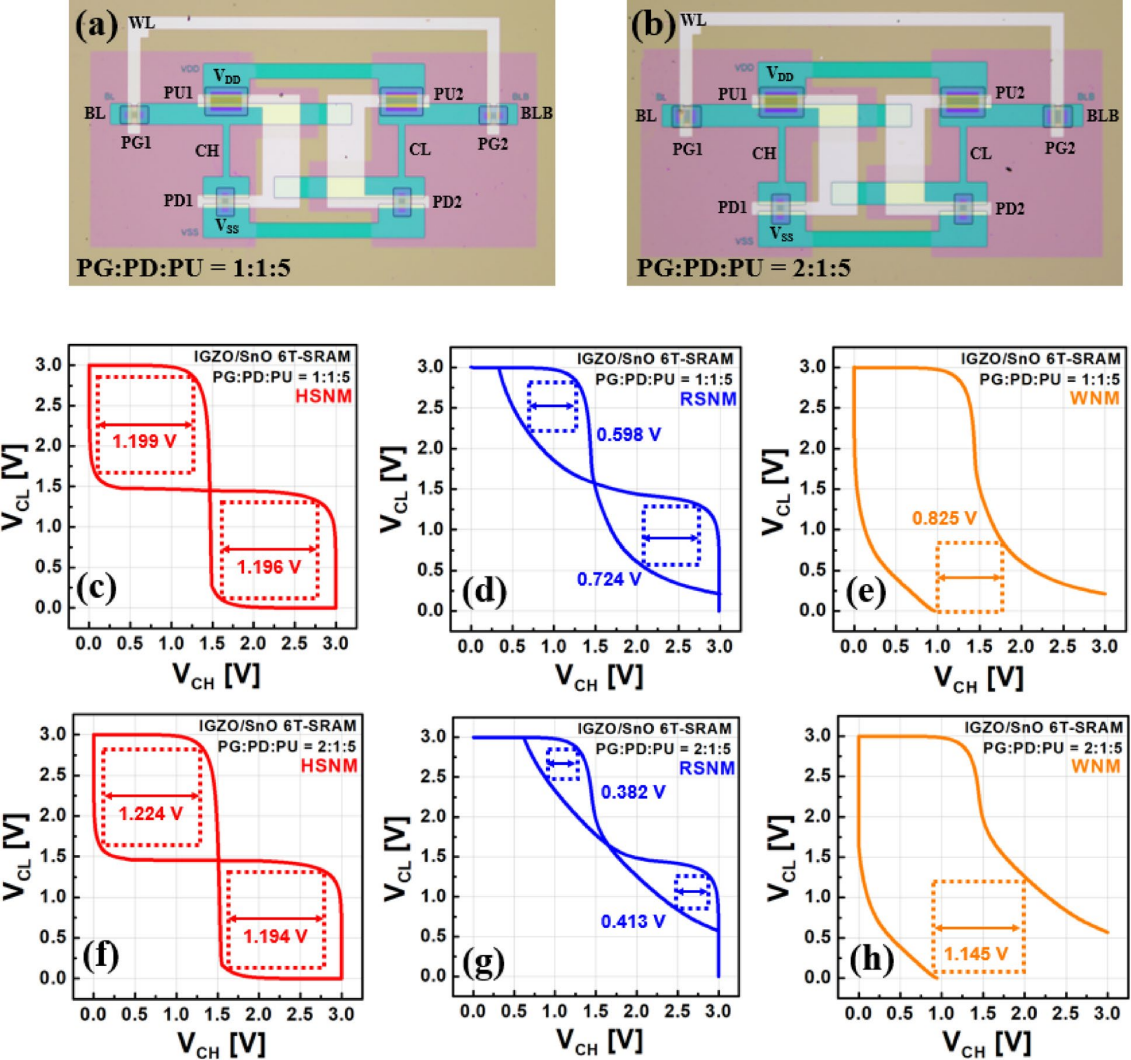
**a**, **b** Optical microscope images of the fabricated IGZO/SnO-based 6 T-SRAM cells with different PG:PD:PU strength ratios of 1:1:5 and 2:1:5, respectively. **c**, **f** Hold static noise margin (HSNM), **d**, **g** read static noise margin (RSNM), and **e**, **h** write noise margin (WNM) characteristics for the two configurations, showing the trade-off between read stability and write ability depending on the transistor strength ratio

For the case with a PG:PD:PU strength ratio of 1:1:5 (Fig. 8a), where the widths of the PG and PD transistors were 30 μm and the PU transistor was 150 μm, the measured HSNM was 1.196 V, RSNM was 0.598 V, and WNM was 0.825 V (Fig. 8c–e). This configuration provided relatively balanced read and write characteristics.

In contrast, when the PG:PD:PU strength ratio was adjusted to 2:1:5 by increasing the PG width to 60 μm (Fig. 8b), the HSNM was 1.194 V, RSNM was reduced to 0.382 V, and WNM increased to 1.145 V (Fig. 8f–h). The widened PG degraded read stability due to reduced cell ratio and pull-up ratio, but it significantly improved write performance.

These results demonstrate that the noise margin characteristics of oxide semiconductor-based SRAM cells can be effectively tuned by adjusting the PG, PD, and PU strength ratios. Notably, this work represents one of the first implementations of IGZO/SnO-based 6 T-SRAM, providing a systematic analysis of noise margin behavior and design optimization guidelines for such devices.

## Conclusions

In this work, we have successfully demonstrated low-voltage, hysteresis-free complementary logic circuits and memory cells based on n-type IGZO TFTs and p-type SnO TFTs with exceptional performance characteristics. Through systematic IGZO channel thickness engineering, we precisely tuned the threshold voltage to optimize inverter switching behavior, achieving a remarkably high voltage gain of up to 146.6 V/V at V_DD_ = 3 V, which is among the highest reported values for oxide-based complementary inverters operating at such low supply voltages. The fabricated circuits exhibited extremely low static power consumption, with maximum power values in the nanowatt range (1.39–311.4 nW), making them ideally suited for ultra-low-power applications including IoT devices and energy harvesting systems.

The implementation of SU-8 passivation layers effectively suppressed bias-stress-induced degradation in both IGZO and SnO TFTs, significantly enhancing long-term operational stability and reducing cycle-to-cycle variation in inverter switching characteristics. Using these optimized devices, we successfully implemented a 3-stage ring oscillator that demonstrated stable oscillations across multiple operating voltages, confirming the robustness and reliability of the complementary circuit design. Furthermore, we fabricated and characterized one of the first IGZO/SnO-based 6 T-SRAM cells, systematically analyzing their static noise margin characteristics and revealing that read/write stability can be effectively tuned by strategically adjusting the pass-gate, pull-down, and pull-up transistor strength ratios.

The comprehensive electrical, structural, and chemical characterization confirmed that both material systems maintain excellent stability and reproducibility after thermal treatment, with minimal hysteresis and superior device uniformity. The demonstrated performance metrics, including rail-to-rail output swing, high noise margins, and ultra-low power consumption, establish this IGZO/SnO platform as a compelling solution for next-generation electronic systems.

Overall, the proposed IGZO/SnO complementary platform offers a highly promising pathway toward the realization of low-power, high-gain, and exceptionally reliable oxide–semiconductor-based logic and memory circuits. These groundbreaking results not only advance the fundamental understanding of complementary oxide semiconductor devices but also pave the way for practical integration with emerging technologies, including non-volatile memory architectures, neuromorphic computing systems, and energy-efficient on-device artificial intelligence hardware. The demonstrated compatibility with low-temperature processing and flexible substrates further opens new opportunities for ubiquitous computing applications in wearable electronics, biomedical devices, and large-area sensor networks, ultimately enabling the next generation of flexible, transparent, and energy-efficient electronic systems.

## Data Availability

The datasets used and/or analysed during the current study are available from the corresponding author on reasonable request.
